# WNT1 Inducible Signaling Pathway Protein 1 Is a Stroma-Specific Secreting Protein Inducing a Fibroblast Contraction and Carcinoma Cell Growth in the Human Prostate

**DOI:** 10.3390/ijms231911437

**Published:** 2022-09-28

**Authors:** Kang-Shuo Chang, Syue-Ting Chen, Hsin-Ching Sung, Shu-Yuan Hsu, Wei-Yin Lin, Chen-Pang Hou, Yu-Hsiang Lin, Tsui-Hsia Feng, Ke-Hung Tsui, Horng-Heng Juang

**Affiliations:** 1 Department of Anatomy, College of Medicine, Chang Gung University, Kwei-Shan, Taoyuan 33302, Taiwan; 2 Graduate Institute of Biomedical Sciences, College of Medicine, Chang Gung University, Kwei-Shan, Taoyuan 33302, Taiwan; 3 Department of Urology, Chang Gung Memorial Hospital-Linkou, Kwei-Shan, Taoyuan 33302, Taiwan; 4 Department of Internal Medicine, Chang Gung Memorial Hospital-Linkou, Kwei-Shan, Taoyuan 33302, Taiwan; 5 School of Nursing, College of Medicine, Chang Gung University, Kwei-Shan, Taoyuan 33302, Taiwan; 6 Department of Urology, Shuang Ho Hospital, New Taipei City 235041, Taiwan; 7 TMU Research Center of Urology and Kidney, Department of Medicine, College of Medicine, Taipei Medical University, Taipei 11031, Taiwan

**Keywords:** prostate, WISP1, stroma, contraction, proliferation, tumorigenesis, EMT

## Abstract

The WNT1 inducible signaling pathway protein 1 (WISP1), a member of the connective tissue growth factor family, plays a crucial role in several important cellular functions in a highly tissue-specific manner. Results of a RT-qPCR indicated that WISP1 expressed only in cells of the human prostate fibroblasts, HPrF and WPMY-1, but not the prostate carcinoma cells in vitro. Two major isoforms (WISP1v1 and WISP1v2) were identified in the HPrF cells determined by RT-PCR and immunoblot assays. The knock-down of a WISP1 blocked cell proliferation and contraction, while treating respectively with the conditioned medium from the ectopic WISP1v1- and WISPv2-overexpressed 293T cells enhanced the migration of HPrF cells. The TNFα induced WISP1 secretion and cell contraction while the knock-down of WISP1 attenuated these effects, although TNFα did not affect the proliferation of the HPrF cells. The ectopic overexpression of WISP1v1 but not WISP1v2 downregulated the N-myc downstream regulated 1 (NDRG1) while upregulating N-cadherin, slug, snail, and vimentin gene expressions which induced not only the cell proliferation and invasion in vitro but also tumor growth of prostate carcinoma cells in vivo. The results confirmed that WISP1 is a stroma-specific secreting protein, enhancing the cell migration and contraction of prostate fibroblasts, as well as the proliferation, invasion, and tumor growth of prostate carcinoma cells.

## 1. Introduction

The CCN family, a group of matrix-cellular proteins in mammals, is comprised of six homologous members that play important roles in the development, inflammation, and tissue repair, and engages in a broad range of pathological processes including fibrosis and cancer [1]. In addition, it has been suggested that the CCN family proteins serve a coordinating role in the tumor microenvironment [2]. The ability of CCNs to bind a great deal of receptors in various cell types may account for the remarkable versatility of their functions, and underscore the diverse signaling pathways that mediate their activities [3]. The Wnt1-inducible signaling protein 1 (WISP1), a member of the CCN family, governs cellular protection, stem cell proliferation, and tissue regeneration in certain disorders [4,5,6,7]. Particularly in prostate, recent studies have suggested that WISP1 is correlated to the tumor growth and metastasis, and the regulation of inflammation in diverse benign diseases [8].

At present, four isoforms of human WISP1 are produced by alternative splicing. The isoform 1 (NM_003882; WISP1v1), chosen as the “canonical” sequence, has 376 amino acids (Aa) with 40.3 kDa. The isoform 2 (NM_0800383, WISP1v2), also known as WISP1v, has 280 Aa with 30.7 kDa without 118-204 Aa and 117 Aa shift from Q to H [9]. The isoform 3 (NM_001204869) misses 24–268 Aa. The isoform 4 (NM_001204870) lacks three internal exons in the coding region (misses 156–367 Aa) and has different amino acids from isoform 1 in 117–155 Aa. Previous investigations have revealed that WISP1 isoforms are overexpressed in scirrhous gastric carcinoma, pancreatic carcinoma, cholangiocarcinoma, and hepatocellular carcinoma [9,10,11,12,13,14,15]. A recent study illustrated another WISP1 isoform (KC344835; WISP1-AS1) which contains 2922 nucleotides long with coding overlaps of parts of the fourth intron and the fifth exon of the human WISP1 gene. WISP1-AS1, regarded as a long non-coding RNA (lncRNA), expressed at higher levels in renal cell carcinoma (RCC) cell lines compared to the primary proximal tubule cells, as well as in the RCC lesions than in the adjacent healthy control tissues from the same patient [16].

An aberrant WISP1 expression is associated with various disorders such as fibrosis and cancer. The role of tumor progression makes WISP1 as an emerging tumor marker [17]. Previous studies have concluded that WISP1 can be deemed as an oncogene in breast, colorectal, liver, oral, glioblastoma, and pancreatic cancers in vitro and in vivo [18,19,20,21,22,23,24]. Although the functions of WISP1 in the urinary tract system have not been well defined, long-term exposure of human primary proximal tubule cells with an ochratoxin A induced WISP1 expression via an ERK1/2-dependent manner [25]. A mutant analysis indicated that patients with urothelial carcinoma cells (UCC) carrying the rs2977530 genetic variants (AG + GG) have a higher risk of developing a more invasive tumor stage and larger tumor suggesting that WISP1 polymorphisms may serve as a marker or a therapeutic target in UCC [26]. Either clinico-pathologic or xenograft animal studies showed that WISP1 is the oncogene marker which may be associated with the epithelial-mesenchymal transition (EMT) and inflammatory microenvironment in human prostate cancer development [8,27]. However, the expression and function of WISP1 in prostate stroma and carcinoma cells in vitro and in vivo have yet to be elucidated.

In this study, we aimed to address the expression and function of WISP1 in the human prostate cells in vitro and to assess the mechanisms of proliferation, migration, and contraction, as well as the invasion activity of WISP1 in human prostate cells in vitro and in vivo.

## 2. Results

### 2.1. Effect of WNT1 Inducible Signaling Pathway Protein 1 (WISP1) on the Proliferation of Human Prostate Fibroblast HPrF Cells

**Figure 1:**
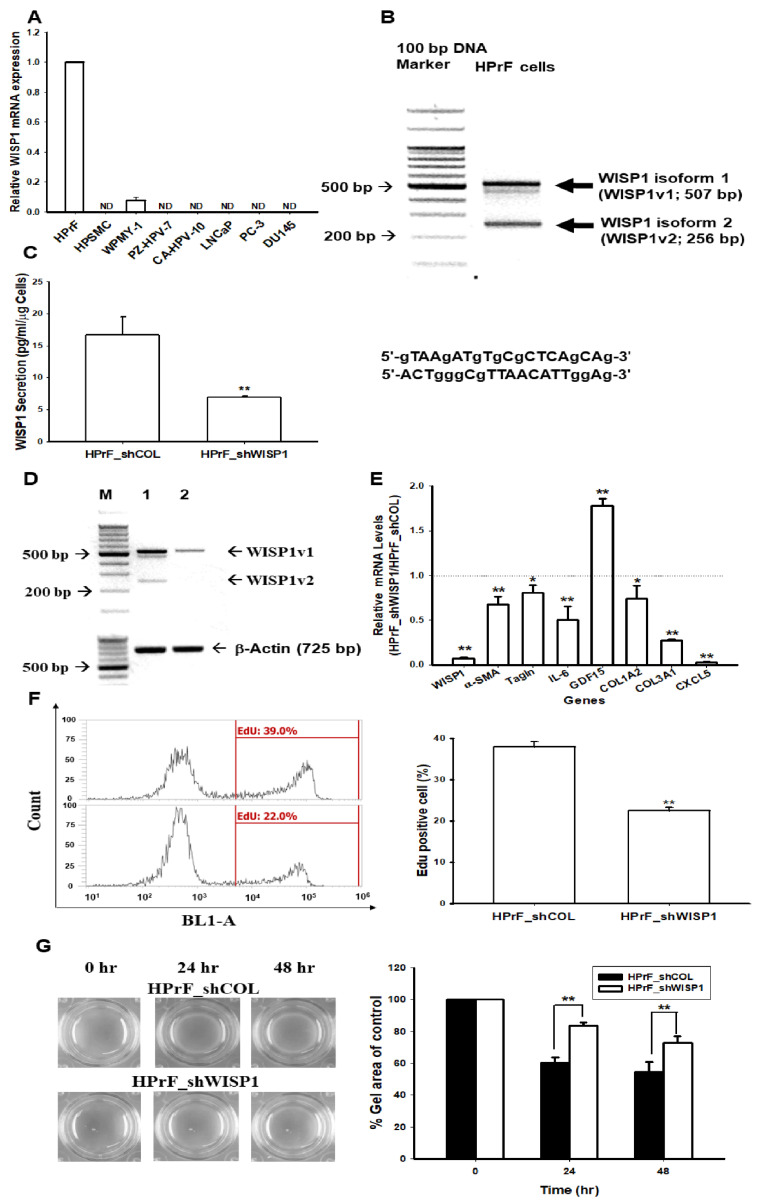
Modulating effect of WISP1 on the cell proliferation and contraction in prostate fibroblast HPrF cells. (**A**) Expression of WISP1 in prostate cells was determined by a RT-qPCR. (**B**) Two WISP1 isoforms (WISP1v1 and WISP1v2) were found in the human prostate fibroblast HPrF cells by RT-PCR with the pair of primers as shown. The secretion of WISP1 (**C**) and mRNA levels of WISP1v1 and WISP1v2 (**D**) after WISP1 was knock-downed in the HPrF cells (M: 100 bp Marker; 1: HPrF_shCOL; 2: HPrF_shWISP1). (**E**) Gene profile of human prostate fibroblast HPrF cells after WISP1 was knock-downed. Cell proliferation (**F**) and contraction (**G**) of the mock-knock-down HPrP (HPrF_shCOL) and the WISP1-knock-down HPrF (HPrF_shWISP1) cells were measured by the EdU proliferation and collage contraction assays, respectively. Data are presented as the mean percentage (±SE; n = 3) of the HPrF_shWISP1 cells in relation to the HPrF_shCOL cells. * *p* < 0.05; ** *p* < 0.01; ND: no detectable.

To determine whether WISP1 expresses in the human prostate stroma or carcinoma cells, we performed RT-qPCR assays using the probes (Hs04234730-m1) for WISP1 which detected all isoforms of WISP1 in the gene bank. Figure 1A illustrated that WISP1 mRNA only expressed in the HPrF and WPMY-1 cells, but not the HPSMC and five prostate cancer cells (PZ-HPV-10, CA-HPV-7, LNCaP, PC-3, and DU145). Further PCR assays using the designed primers (5′-GTAAGATGTGCGCTCAGCAG-3′ and 5′-ACTGGGCGTTAACAT TGGA G-3′), found that HPrF cells express two isoforms of WISP1 (WISP1v1 and WISP1v2) mRNA (Figure 1B). The sequences of nucleic acids and predicative proteins of WISP1v1 and WISP1v2 isoforms were confirmed by extensive sequencing (Supplementary Figure S1). We knocked down the WISP1 in HPrF cells to determine the function of WISP1 in cells of human prostate fibroblasts. The results of ELISA (Figure 1C) and PCR (Figure 1D) assays indicated that WISP1 was inhibited in HPrF_shWISP1 cells compared to mock-knock-down HPrF_shCOL cells. Further RT-qPCRs showed the gene profile of HPrF cells after knocking down the WISP1 gene (Figure 1E). Interestingly, the GDF15 gene expressions were upregulated, while the gene expressions of alpha-smooth muscle actin (α-SMA), transgelin (Tagln), collagen type I alpha 2 chain (COL1A2), collagen type III alpha 1 chain (COL3A1), interleukin 6 (IL-6), and C-X-C motif chemokine 5 (CXCL5) genes were significantly downregulated when WISP1 was knock-downed in HPrF cells. Moreover, the knock-down of WISP1 decreased the proliferation (Figure 1F) and contraction (Figure 1G) of HPrF cells which were determined by the EdU proliferation and collagen-based cell contraction assays, respectively.

### 2.2. WISP1 Enhances the Migration of Prostate Fibroblast HPrF Cells

**Figure 2:**
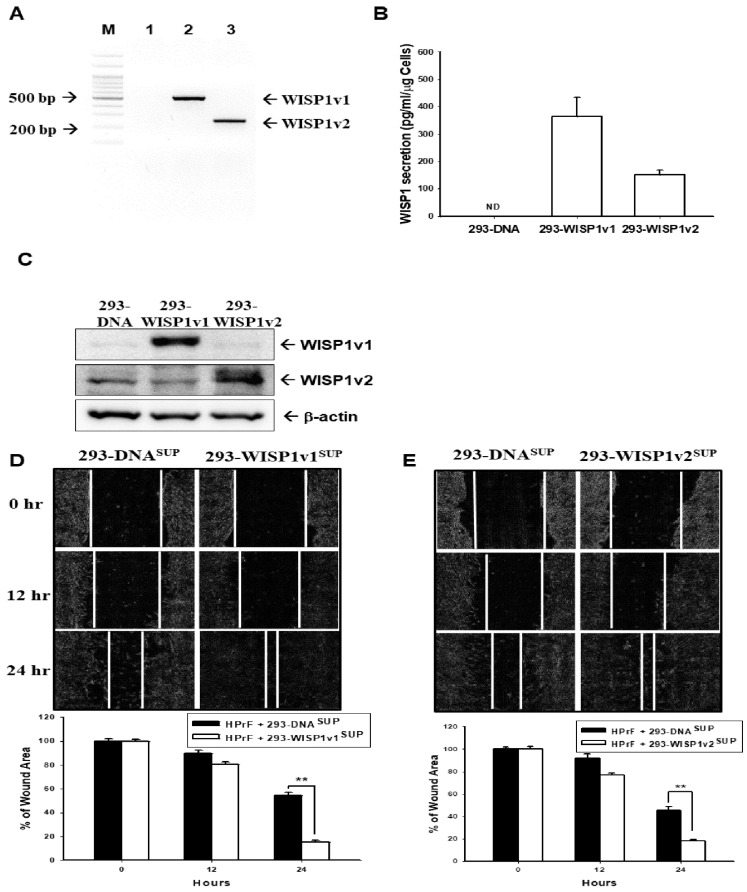
Modulating effect of the conditioned media from the ectopic WISP1 overexpressed-HEK 293 cells on the cell migration in the prostate fibroblast HPrF cells. (**A**) Expressions of WISP1v1 and WISP1v2 after the ectopic-overexpressed WISP1v1 and WISP1v2 in the HEK293 cells were assessed by the RT-PCR (M: 100 bp Marker; 1: HPrF_shCOL; 2: HPrF_shWISP1), ELISA (**B**), and immunoblot (**C**) assays. The abilities of migration in HPrF cells treated with the conditioned media of the ectopic WISP1v1- (**D**) or WISP1v2- (**E**) overexpressed-HEK293 cells. The white line indicated the average of the cellular leading edges and the size of the wound area was calculated by ImageJ. Data are presented as the mean percentage (±SE; n = 3) in relation to the mock-transfected HEK293 (293-DNA) cells. ** *p* < 0.01; ND: no detectable.

We transfected mock-transfected vector (pcDNA3), as well as pcWISP1v1 and pcWISP1v2 expression vectors, respectively, into HEK293 cells to obtain the soluble proteins of WISP1v1 and WISP1v2. The results from the RT-PCR (Figure 2A), ELISA (Figure 2B), and immunoblot (Figure 2C) assays confirmed that WISP1v1 and WISP1v2 proteins were overexpressed in 293-WISP1v1 and 293-WSIP1v2 cells, respectively. However, the commercially available WISP1v1 antibody (ab178547, abcam) has a lower sensitivity of WISP1v2 than WISP1v1, hence the WISP1v2 protein has to be overly exposed to be detected. The overexpressed-WISP1v1 and -WISP1v2 were cultured, respectively, in the serum-free medium for 24 h, and then the media were collected as the conditioned medium. The concentration of WISP1 in conditioned media of both cells was adjusted to 1 ng/mL with the serum-free medium and stored in a −80 °C freezer. The results of the wound healing migration assays revealed that treatments of the 1 mL conditioned medium from the ectopic WISP1v1-overexpressed 293T (Figure 2D) or the ectopic WISP1v2-overexpressed 293T (Figure 2E) cells induced the migration of HPrF cells.

### 2.3. TNFα Induces the WISP1 Secretion to Enhance the Contraction of Human Prostate Fibroblast HPrF Cells

**Figure 3:**
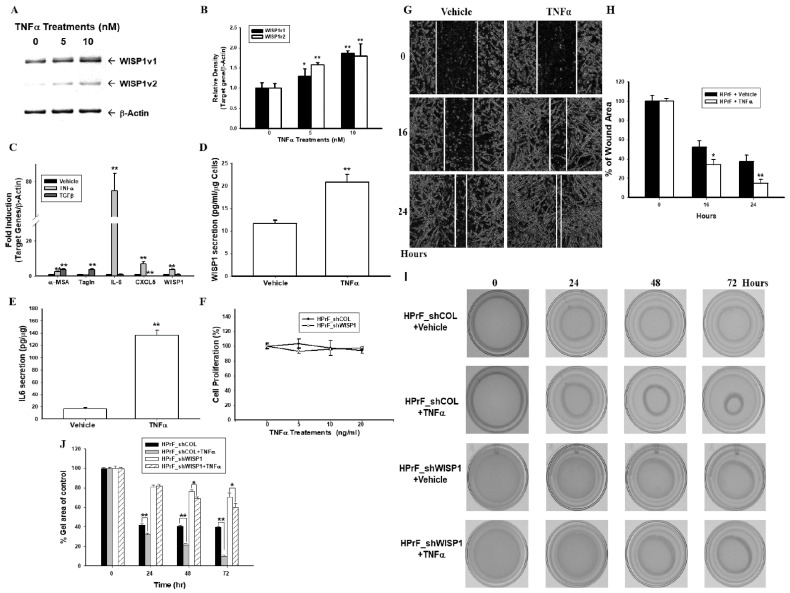
Knock-down of WISP1 attenuates the activation of TNFα on the cell migration and contraction in prostate fibroblast HPrF cells. (**A**) TNFα treatments (0–10 ng/mL) upregulated the WISP1 expression in HPrF cells determined by RT-PCR assays. (**B**)The quantitative analysis was presented as a relative density of WISP1v1 or WISP1v2/β-Actin. (**C**) Gene profile of the human prostate fibroblast HPrF cells after they were treated with 10 ng/mL of TNFα or TGFβ, as indicated. The protein levels of WISP1 (**D**) and IL-6 (**E**) in the conditioned media of HPrF cells treated with/without TNFα. (**F**) The ability of the proliferation of HPrF cells was measured by the CyQUANT cell proliferation assay kit after it was treated with various dosages of TNFα, as indicated, for 48 h. (**G**) The ability of the migration of HPrF cells treated with/without 10 ng/mL of TNFα. The white line indicated the average of the cellular leading edges and the size of the wound area was calculated by ImageJ. (**H**) Data are presented as the mean percentage (±SE; n = 4) in relation to the vehicle-treated HPrF cells. (**I**) The ability of the contraction of HPrF_shCOL and HPrF_shWISP1 cells treated with/without 10 ng/mL of TNFα. (**J**) Data are presented as the mean percentage (±SE; n = 3) of gel area in relation to the time 0. * *p* < 0.05; ** *p* < 0.01.

The RT-PCR assays (Figure 3A) and the quantitative analysis (Figure 3B) showed that the TNFα treatments enhanced the mRNA levels of WISP1v1 and WISP1v2. Further RT-qPCR assays exhibited that only 10 ng/mL of TNFα but not TGFβ1 treatments enhanced the WISP1 expressions (Figure 3C). The TNFα treatment also induced the CXCL5 gene expression, while the TGFβ1 treatment upregulated the α-SMA and Tagln gene expressions. The results of ELISA confirmed that TNFα induced both the IL-6 and WISP1 secretions (Figure 3D,E). Although the results of the CyQUANT cell proliferation assays showed that TNFα did not affect the proliferation of HPrF cells (Figure 3F), the wound healing assays and the quantitative analysis revealed that TNFα induced the migration of HPrF cells (Figure 3G,H). Further collagen-based cell contraction assays demonstrated that the 10 ng/mL TNFα treatment induced the cell contraction while the knock-down of WISP1 attenuated this response of the HPrF cells (Figure 3I). The quantitative analysis of the cell contraction was presented in the Figure 3J.

### 2.4. Ectopic Overexpression of WISP1v1 but Not WISP1v2 Enhances Cell Proliferation

**Figure 4:**
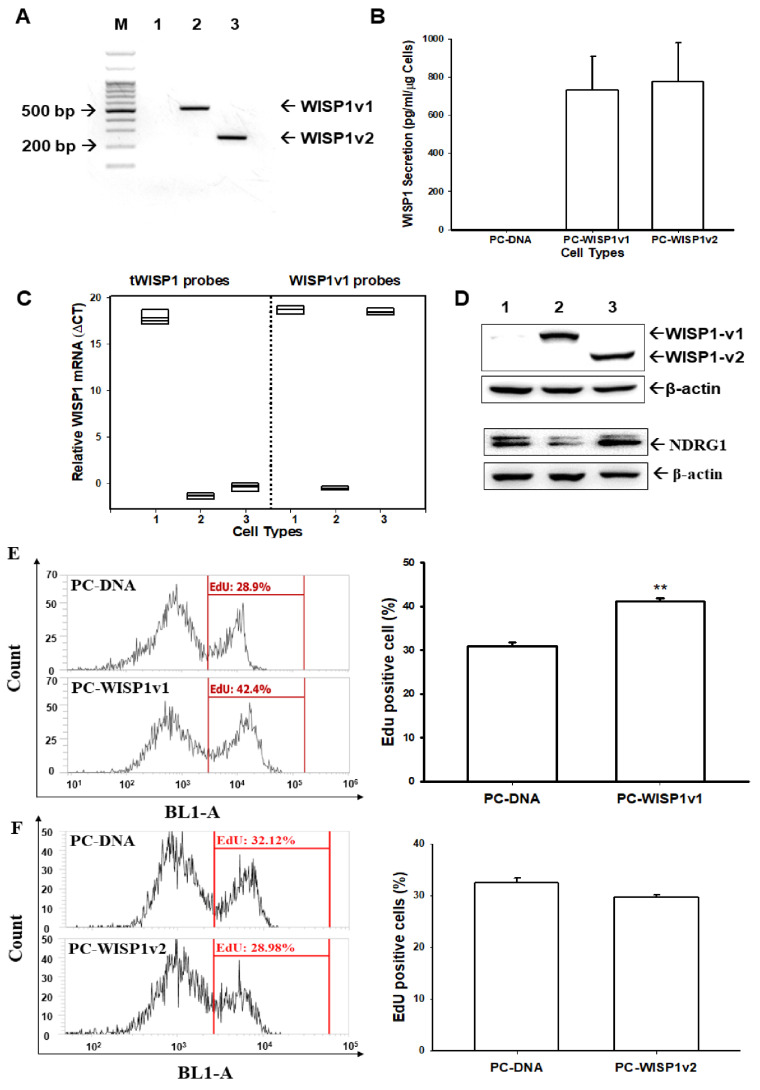
Modulating effect of WISP1v1 and WISP1v2 on the cell proliferation in prostate carcinoma PC-3 cells. Expressions of WISP1v1 and WISP1v2 after the ectopic-overexpressed WISP1v1 and WISP1v2 in the PC-3 cells were assessed by RT-PCR (**A**), ELISA (**B**), and RT-qPCR (**C**) assays. (**D**) The expressions of WISP1v1, WISP1v2, NDRG1, and β-actin were determined by immunoblot assays (M: 100 bp Marker; 1: PC-DNA; 2: PC-WISP1v1; 3: PC-WISP1v2). The abilities of the proliferation of PC-DNA, PC-WISP1v1 (**E**), and PC-WISP1v2 (**F**) cells were measured by EdU flow cytometry assays. Data are presented as the mean percentage of the EdU positive cells (±SE; n = 4). ** *p* < 0.01.

To evaluate the effect of WISP1 on the prostate carcinoma cells, we constructed the ectopic-overexpressed prostate carcinoma PC-3 cells by stably transfecting the pcWISP1v1 and pcWISP1v2 expression vectors, respectively, into PC-3 cells. The results of the RT-PCR (Figure 4A), ELISA (Figure 4B), RT-qPCR (Figure 4C), and immunoblot (Figure 4D) assays confirmed that the stable clones of PC-WISP1v1 and PC-WISP1v2 specifically overexpressed WISP1v1 and WISP1v2. Interestingly, further immunoblot assays indicated that only the ectopic overexpression of WISP1v1 downregulated the protein levels of NDRG1 in PC-3 cells (Figure 4C). Additionally, the EdU flow cytometry assays revealed that the ectopic-overexpressed WISP1v1 enhanced the proliferation of PC-3 cells (Figure 4E,F).

### 2.5. Ectopic Overexpression of WISP1v1 but Not WISP1v2 Enhances the Cell Migration via the Induction of the Epithelial-Mesenchymal Transition

**Figure 5:**
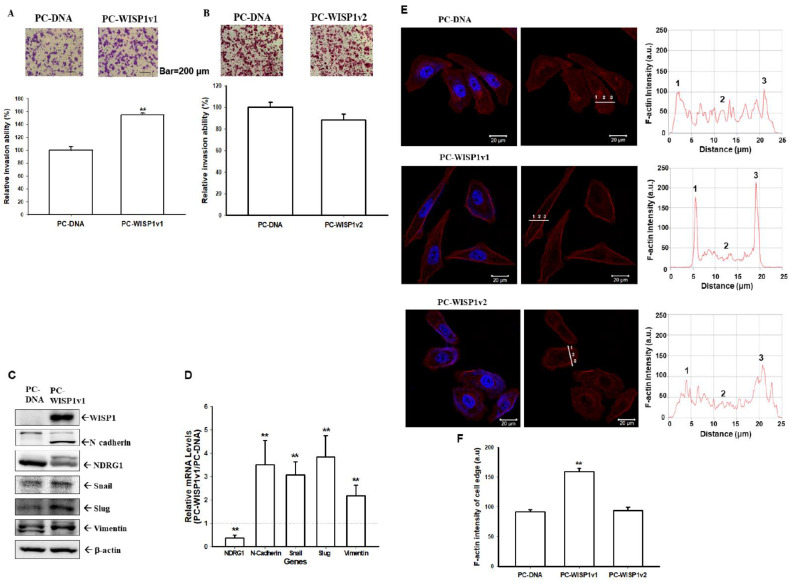
Modulating effect of WISP1v1 on the cell invasion in the prostate carcinoma PC-3 cells. The invasion ability of PC-3 cells after the ectopic overexpression of WISP1v1 (**A**) and WISP1v2 (**B**) was determined by in vitro Matrigel invasion assays. (**C**) The protein levels of NDRG1 and EMT markers (N-cadherin, snail, slug, vimentin) were determined by immunoblot assays. (**D**) The mRNA levels of the NDRG1 and EMT markers were determined by RT-qPCR. Data were presented as target genes/β-Actin of PC-WISP1v1 cells relative to PC-DNA cells. (**E**) The F-actin staining with Texas Red X-Phalloidin and the immunofluorescence was recorded using a confocal microscope. (**F**) The intensities were measured along the line from the peripheral to the central of the cells, and the quantitative analysis of the F-actin fluorescence intensity of PC-DNA, PC-WISP1v1, and PC-WISP1v2 cells (±SE, *n* = 4). ** *p* < 0.01.

The Matrigel invasion assays showed that the ectopic overexpression of WISP1v1 (Figure 5A) but not WISP1v2 (Figure 5B) significantly increased the invasion of PC-3 cells. Further immunoblot (Figure 5C) and RT-qPCR assays (Figure 5D) verified that the ectopic overexpression of WISP1v1 induced the expressions of N-cadherin, snail, slug, and vimentin; in the contrary, the ectopic overexpression of WISP1v1 blocked the NDRG1 expression. Since immunoblot and RT-qPCR assays confirmed that the WISP1v1 overexpression upregulated the markers of the EMT, we used an immunofluorescence image to assess the distribution of the F-actin of PC-DNA, PC-WISP1v1, and PC-WISP1v2 cells. Figure 5E showed the fluorescence images of the F-actin in cells indicating the distinct differences in the F-actin distribution among PC-DNA, PC-WISP1v1, and PC-WISP1v2 cells stained with Texas Red X-Phalloidin. The different densities of the F-actin were analyzed based on the quantification of the fluorescence intensity using Zen blue edition software. The results of the quantitative analysis revealed that the F-actin in the WISP1v1-overexpressed but not the WISP1v2-overexpressed PC-3 cells was distributed mainly on the periphery. Collectively, these results suggested that the overexpression of WISP1v1 in PC-3 cells significantly enhanced the cell invasion via the upregulation of the EMT.

### 2.6. Ectopic Overexpression of WISP1v1 Enhances Tumor Growth in a Xenograft Animal Model

**Figure 6:**
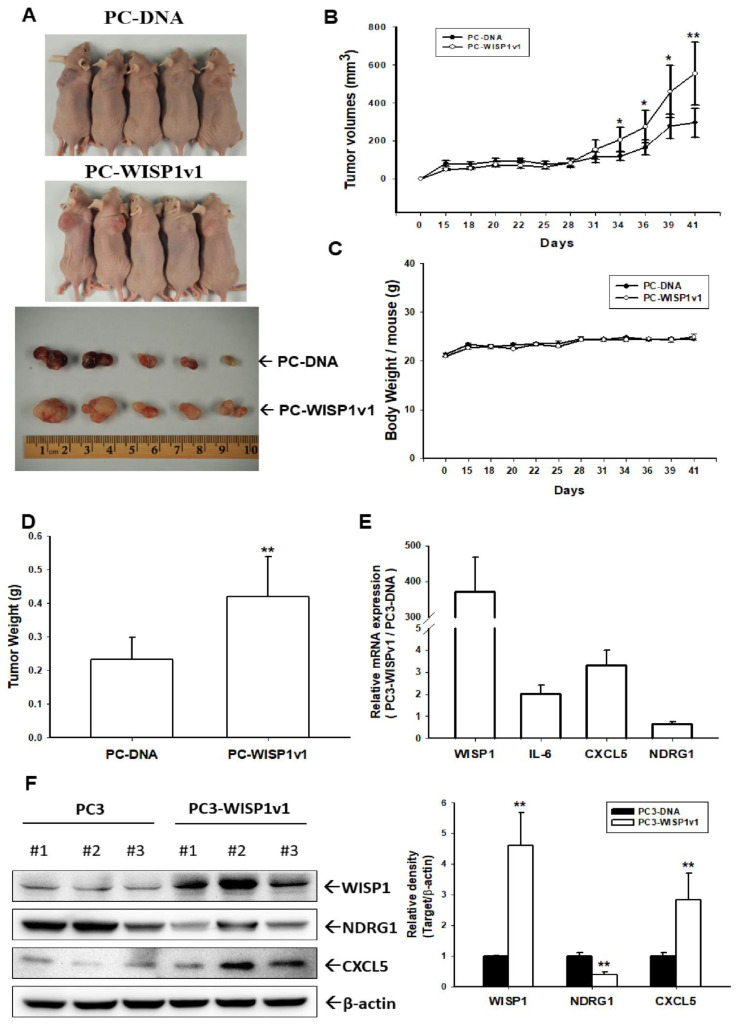
Modulating effect of WISP1v1 on the tumor growth of prostate carcinoma PC-3 cells. (**A**, top) PC-DNA and PC-WISP1v1 cells were injected subcutaneously in the dorsal area of the four week old male athymic nude mice (n = 5). (**A**, bottom) Tumors from the PC-DNA and PC-WISP1v1 cells were recorded after the mice were sacrificed. The volumes of tumor (**B**) and body weight (**C**) were measured every 2–3 days during a period of 41 days. (**D**) The tumor weights (±SE; n = 5) were recorded after the sacrifice. (**E**) The mRNA levels (±SE; n = 3) of WISP1, IL-6, CXCL5, and NDRG1 were determined by RT-qPCR. The protein levels of WISP1, NDRG1, CXCL5, and β-Actin (±SE; n = 3) of the tumors from the PC-DNA and PC-WISP1v1 cells were determined by immunoblot assays (**F**, left). The quantitative analysis was presented as the relative density of the target proteins/β-Actin (**F**, right). * *p* < 0.05, ** *p* < 0.01.

In order to extend the effect of WISP1v1 on the cell proliferation in vitro, we continued to evaluate the effect of WISP1v1 on tumor growth in vivo. The PC-DNA and PC-WISP1v1 cells were xenografted into male nude mice (BALB/cAnN-Foxn1). The mice were sacrificed and the tumors were collected on day 41 after inoculation (Figure 6A). The results of the xenograft study showed that the tumors in the PC-WISP1v1 group grew faster in comparison to the PC-DNA group during the 41 days of the experimental period (Figure 6B). The final tumor volume was 295.97 ± 66.12 and 554.65 ± 134.75 mm^3^ for the PC-DNA and PC-WISP1v1 cells, respectively, after 41 days of inoculation. However, the average body weight between the PC-DNA and PC-WISP1v1 groups was not significantly different (Figure 6C). The average tumor weight was increased in the PC-WISP1v1 group compared to that in the PC-DNA group (0.23 ± 0.07 vs. 0.45 ± 0.11 g; Figure 6D). These results suggested that the ectopic overexpression of WISP1v1 in PC-3 cells significantly enhanced the tumorigenesis in the xenograft mice model. Three tumors from the xenograft tumors of both the PC-DNA and PC-WISP1v1 groups were extracted to determine the protein and mRNA levels after the mice were sacrificed. The mRNA levels of WISP1v1, IL-6, and CXCL5 were upregulated in the xenograft tumors derived from the PC-WISP1v1 cells compared to those derived from the PC-DNA cells (Figure 6E). Further immunoblot assays revealed that the protein levels of WISP1v1 and CXCL5 were increased in the xenograft tumors derived from the PC-WISP1v1 cells compared to those from the PC-DNA cells. However, both the mRNA and protein levels of NDRG1 were decreased in the xenograft tumors derived from the PC-WISP1v1 cells compared to those from the PC-DNA cells (Figure 6F).

## 3. Discussion

WISP1 is a matricellular protein and belongs to the CCN family which plays various biological roles in the pathological processes [1]. At least four isoforms of human WISP1 have been identified in situ by alternative splicing and found to be overexpressed in several cancers [17]. The WISP1-AS1 isoform is a lncRNA which has been suggested as the tumor marker of renal cell carcinoma and colorectal cancer [16,28]. In the present study, we confirmed that WISP1 only expresses in the human prostate stromal cells, HPrF and WPMY-1, but not the prostate carcinoma cells (Figure 1). A recent clinicopathological study verified that the WISP1 expression was significantly higher in the stroma when compared with the epithelium in prostate cancer tissues [8]. Using the specific pair primers, our RT-PCR results identified two isoforms of WISP1 expressed in the HPrF cells. One is the WISPv1 (NM_003882) with a full-length canonical coding protein and another is the WISP1v2 (NM_0800383) which missed the 260 nucleotides of exon 3 that encode the VWC module sequences in the wild type of mRNA species. Both isoforms have been identified in earlier studies [9,11]. To our knowledge, this is the first study identifying these two isoforms in human prostate stroma cells, although the potential functions of WISP1 isoforms have not been explored yet in the prostate.

An early review study has indicated that the autocrine and paracrine of stromal cells affect the tumor growth of the prostate [29]. In the present study, we revealed that both knock-downed WISP1v1 and WISP1v2 expressions attenuated the cell proliferation and contraction of the human prostate fibroblasts, HPrF cells, in vitro, which is in agreement with a previous study in which recombinant WISP1 treatments increased the cell proliferation of mouse and human lung fibroblasts [30]. Treatments of the conditioned media from the ectopic overexpression of WISP1v1 and WISP1v2, respectively, also induced the migration ability of HPrF cells in vitro (Figure 2). These results are in accordance with a previous study that verified that WISP1 works on a α5β1 integrin receptor to stimulate the cell migration of human dermal fibroblasts [31]. Interestingly, the knock-down of WISP1 downregulated the mRNA levels of α-SMA, Tagln, IL-6, CXCL5, COL1A2, and COL3A1 (Figure 1E). The COL3A1, one of the 93 stroma-derived metastasis signature genes, was identified from the metastatic primary prostate cancer [32]. The results suggested that WISP1 may induce pro-inflammatory cytokines and chemokines, the expression of α-SMA, as well as other contractile proteins involving the migration and contraction of stroma cells [33,34,35,36,37].

The overexpression of WISP1 has been described in multi-organ fibrosis and tissue remodeling [38]. Early studies have verified that WISP1 enhances the proliferation of fibroblast and smooth muscle cells in the pulmonary tract [33,39,40]. The present study, used the conditioned media from the ectopic overexpression of WISP1v1 and WISP1v2, respectively. Our study clearly demonstrated that both WISP1v1 and WISP1v2 induced the cell migration of HPrF in vitro (Figure 2). The results are consistent with other studies that revealed that WISP1 enhances the cell migration and proliferation in the asthmatic mouse bronchial smooth muscle of the remodeling airway and the vascular smooth muscle cells [7,23,33,40].

In the present study, we also found that the TNFα but not the TGFβ induced expressions and secretions of WISP1v1 and WISP1v2, respectively, in HPrF cells (Figure 3A), although reports have indicated that WISP1 is a downstream gene of TGFβ in the studies of primary lung fibroblasts and liver fibrosis [36,40,41]. Our study confirmed that TGFβ induced the mRNA levels of α-SMA and Tagln which is similar to other studies in human skeletal stem cells, rat lung fibroblasts, and human scleral fibroblasts [34,35,42]. The results of Figure 3 indicated that 10 ng/mL of the TNFα treatment upregulated the expressions of α-SMA, IL-6, CXCL5, and WISP1 in HPrF cells, which is consistent with other studies in various cells [33,38,40,43]. Our study revealed that TNFα induced WISP1, pro-inflammatory cytokines and chemokines, and α-SMA to enhance the cell migration and contraction. Interestingly, the TNFα treatment induced the migration of HPrF cells; yet, the knock-down of WISP1 blocked the activation of TNFα on the contraction of the HPrF cells in vitro, although the TNFα treatments did not affect the proliferation of the HPrF cells. Taking these together, our studies verified that the induction of TNFα in the expressions of WISP1v1 and WISP1v2 enhanced the cell migration and contraction of the human prostate fibroblasts in vitro.

Recent reports have proposed that cancer-associated fibroblasts or the fibroblast heterogeneity has been implied concerning the tumor growth in several cancers [44,45]. The tumor microenvironment, namely the epithelial-stromal crosstalk, during the initiation progression and the metastatic potential of various cancers including prostate cancer [46,47]. The stromal gene expression also has been suggested to predict the metastatic primary prostate cancer [32]. WISP1 has been detected mainly in the tumor stroma of prostate tissues in two immunohistochemistry studies from independent laboratories [8,27]. Another study showed that the osteoblast-derived WISP1 enhanced the proliferation and invasion of prostate carcinoma cells in vitro [48]. Our study agreed with the results that suggested that WISP1 may act as a paracrine secretion factor to modulate the oncogenic characteristics of prostate carcinoma cells. Although WISP1v2 has been regarded as the tumor marker in scirrhous gastric carcinoma and cholangiocarcinoma in vivo [9,11]. Our present study found that the ectopic overexpression of WISP1v1 but not WISP1v2 enhanced the proliferation and invasion of prostate carcinoma PC-3 cells via the downregulation of NDRG1 (Figure 4 and Figure 5). NDRG1 was found as the downstream gene of WISP1 in the studies on the breast cancer cells [20,49]. These results suggested that WISP1v2 might not have the oncogene characters in the prostate carcinoma cells in vitro. The results of immunoblot and RT-qPCR assays confirmed that the ectopic overexpression of WISP1v1 modulated the expressions of the EMT markers including N-cadherin, slug, snail, and vimentin (Figure 5C,D). Further F-actin staining and a quantitative analysis also suggested that the ectopic overexpression of WISP1v1 but not WISP1v2 significantly reorganized the actin filaments from thin cortical bundles to thick, parallel, contractile bundles in the human prostate carcinoma PC-3 cells in the epithelial-mesenchymal transition (EMT) process (Figure 5F). A recent study has indicated a correlation of the mRNA expression between WISP1 and the EMT in prostate adenocarcinoma tissues [8]. Other in vitro studies also illustrated that WISP1 promoted the EMT in oral squamous cell carcinoma cells and melanoma cells [50,51].

An early study using the specific anti-WISP1 antibody indicated that WISP1 enhanced prostate cancer growth and bone metastasis in animal models [27]. Our xenograft animal studies showed that the ectopic overexpression of WISP1v1 increased the tumor growth in vivo. The protein and mRNA levels of the tumor derived from PC-WISP1v1 cells in the xenograft animal model hat indicated that the WISP1 induced CXCL5 but downregulated NDRG1 in vivo (Figure 6). The results of the downregulation on NDRG1 by the ectopic overexpression of WISP1v1 are in agreement with a previous study in breast carcinoma cells [20]. WISP1v1 induced IL-6 and CXCL5 in vivo, suggesting that WISP1v1 might be involved in the pro-inflammatory environment of prostate adenocarcinoma in vivo. However, it should be made aware that using the WISP1 ectopic-overexpressed PC-3 cells in the xenograft study may not truly demonstrate the microenvironment of prostate cancer in vivo. Further translatable approaches to the roles of WISP1 in the human prostate cancer in vivo still need to be explored. In combination with recent clinicopathological studies, we verified that WISP1 may be considered as a potential oncogene crosstalk between stromal and carcinoma epithelial cells in the human prostate cancer [8].

## 4. Materials and Methods

### 4.1. Materials, Cell Lines, and Cell Cultures

The human prostate fibroblast cells and fibroblast medium were purchased from ScienCell (Cat # 4430 and SC-2301; Carlsbad, CA, USA). The human normal prostate smooth muscle cells and PriGrow X medium were purchased from abm (cat #T4079 and TM4079, Richmond, BC, Canada). HEK-293T, PZ-HPV-7, CA-HPV-10, LNCaP, PC-3, and DU145 cells were obtained from the Bioresource Collection and Research Center (BCRC, Hsinchu, Taiwan) and cultured, as described previously [52]. The human prostate stromal myofibroblast WPMY-1 cells were purchased from the American Type Culture Collection (ATCC; CRL-2854; Manassas, VA, USA). The cells were cultured as instructed by the manufacturers. Fetal bovine serum (FBS) was purchased from HyClone Laboratories, Inc. (Logan, UT, USA); RPMI 1640 medium came from Life Technologies (Rockville, MD, USA) and Matrigel came from BD Biosciences (Bedford, MA, USA). TNFα and TGFβ were purchased from PeproTech (Cranbury, NJ, USA).

### 4.2. Gene Knock-down

The cells were plated onto 6-well plates for 24 h, and then the culture medium was replaced with a RPMI-1640 medium plus 10% FBS and 5 μg/mL of polybrene. The cells were transduced with WISP1 shRNA lentiviral transduction particles (sc-39335-V; Santa Cruz Biotechnology, Santa Cruz, CA, USA), as described previously [20]. Two days after transduction, the cells were selected by incubation with 10 μg/mL of puromycin dihydrochloride. The mock-transfected cells were transduced with control shRNA lentiviral particles (sc-108080; Santa Cruz Biotechnology) and selected clonally in the same manner as the gene-knockdown cells.

### 4.3. Expression Vector Constructs and the Stable Transfection

The expression vector of the human WISP1v1 cDNA was cloned, as described previously [20]. The WISP1v2 expression was purchased from Genescript Biotech Corporation. (OHu23245; Piscataway, NJ, USA). The WISP1v1 and WISP1v2 expression vectors were transfected respectively into the human prostate carcinoma PC-3 cells by electroporation, as described previously [53]. The transfected cells were maintained in a RPMI medium with 10% FBS and selected by Zeocin™ salt at 100 µg/mL (Thermo Fisher Scientific Inc., Waltham, MA, USA). The mock-transfected cells were transfected with a control pcDNA3.1 expression vector and were clonally selected in the same manner as the gene-overexpression cells.

### 4.4. Cell Proliferation Assay

The proliferation of the HPrF cells after treatment with various dosages of TNFα, as indicated, was measured by the CyQUANT cell proliferation assay kit (Thermo Fisher Scientific Inc. Vienna, Austria). Briefly, we seeded 3000 cells into each well of a 96-well plate in a RPMI 1640 medium with 10% FBS for 24 h. The cells were inoculated with 0–10 ng/mL of TNFα in a serum free RPMI 1640 medium for another 16 h, and then, washed two times with phosphate buffered saline (PBS). Once the cell pellets were frozen at −80 °C for 24 h, the cell pellets were thawed at room temperature, and then, a 200 μL of the CyQUANT GR dye and a cell lysis buffer were added to each sample. The measurement of the fluorescence of the 488 nm excitation was performed by using the synergy H1 microplate reader (BioTek Instruments, Inc. Santa Clara, CA, USA) after 10 min incubation, as described previously [54]. The proliferation of the PC-3 cells was measured by the EdU flow cytometry assays performed, as previously, using the Click-iT EdU Flow Cytometry Assay Kit (Thermo Fisher Scientific Inc., Waltham, MA, USA). The cells of PC-DNA, PC-WISP1v1, and PC-WISP1v2 were cultured in a RPMI 1640 medium with 10% FBS for 24 h, and then, cultured in a serum-free medium for another 24 h. Once cells were incubated with a full-medium for another 24 h, the EdU (5-ethynyl-2′-deoxyuridine; 10 μM) was added to the culture medium for 2 h. The EdU fluorescence of the 5000–10,000 cells were detected using an Attune NxT acoustic focusing cytometer (Thermo Fisher Scientific Inc., Waltham, MA, USA).

### 4.5. Cell Contraction Assay

The cell contraction was performed by using a cell contraction assay kit (CBA-201, Cell Biolabs, Inc., San Diego, CA, USA). Briefly, cells were collected by using a trypsin-EDTA and then centrifuged at 500× *g* for 5 min. The cell pellets were resuspended in desired medium at 2 × 10^5^ cells/100 μL. A collagen lattice was prepared by mixing 100 μL of the cell suspension and 400 μL of the cold Collagen Gel Working Solution, and then a 500 μL of the cell-collagen mixture was added in a 24-well plate and incubated for 1 h at 37 °C. Following the collagen polymerization, 1.0 mL of the culture medium was added at the top of each collagen gel lattice. The change of collagen gel size was measured every day, as indicated, and photographed with the camera (COOLPIX 8700, Shinagawa, Tokyo, Japan).

### 4.6. Cell Migration

The cell migratory ability of the HPrF cells was performed using a wound-healing assay. The cells were over-grown in the 12-well plates; then, the monolayer cells were uniformly scratched with sterile pipette tips. The cast-off cells were washed away, and then the cells were incubated with 1 mL of the medium, as indicated. The center spot of each well of the plate was marked and measured at different times, as indicated. We recorded the scratched area using the inverted microscope (Olympus 1X71, Tokyo, Japan). Image J software (version 1.52a) was used to measure the scratched area at different periods, as indicated. The cell migratory ability of wound-healing was assessed using the percentage of the wound area (wound area at indicated times to wound area at 0 h).

### 4.7. Immunoblot Assays

Equal amounts (20 μg or 40 μg) of cell extracts were separated on a 10% or 12% dodecyl sulfate polyacrylamide gel electrophoresis (SDS-PAGE) gel. The blotting membranes were probed using antiserum of WISP1 (ab178547, Abcam, Cambridge, UK), N-cadherin (GTX127345, GeneTex, Inc., Irvine, CA, USA), NDRG1 (42-6200, Invitrogen, Carlsbad, CA, USA), Snail (C15D3, Cell Signaling Technology, Inc., Danvers, MA, USA), Slug (C19G7, Cell Signaling Technology, Inc.), Vimentin (AP2739B, Abgent, San Diego, CA, USA), or β-actin (T0022, Affinity bioscience, Cincinnati, OH, USA), and then detected by using the Western Lightning™ Plus Chemiluminescence detection kit (Perkin Elmer, Inc., Waltham, MA, USA). The band intensities were recorded by the LuminoGraph II (Atto Corporation, Tokyo, Japan) and the data was analyzed with the ImageJ.

### 4.8. Reverse Transcription-Polymerase Chain Reaction (RT-PCR) and the Quantitative RT-qPCR

The total RNA was extracted from cells with a Trizol reagent and the cDNAs were synthesized using the superscript III preamplification system. The PCR reaction was carried out in the Biometra T3000 thermocycler (Jena, Germany) and the PCR products were separated with 2% agarose gel electrophoresis. The real-time PCR (qPCR) was performed using a CFX Connect Real-PCR system (Bio-Rad Laboratories, Foster City, CA, USA). TaqMan™ gene expression master mix and polymerase chain reaction (PCR) FAM dye-labeled TaqMan MGB probes for human WISP1 (Hs04234730_m1 for total isoforms and Hs00180245 for WISP1v1), α-SMA (Hs00426835_g1), Tagln (Hs01038777_g1), IL-6 (Hs00985639_m1), GDF15 (Hs00171132_m1), COL1A2 (Hs01028956_m1), COL3A1 (Hs00943809), CXCL5 (Hs01099660_g1), NDRG1 (Hs00608387_m1), N-cadherin (Hs00169953_m1), snail (Hs00195591_m1), slug (Hs00161904_m1), vimentin (Hs00185584_m1), and β-actin (Hs01060665_g1) were purchased from Thermo Fisher Scientific Inc. (Vilnius, Lithuania). The mean cycle threshold (Ct) values were calculated for β-actin and the target genes using Bio-Rad CFX manager 3.1 (Bio-Rad Laboratories, Foster City, CA, USA). The Ct values for the target genes were normalized against the β-actin control probe to calculate the Δ Ct values as, described previously [55].

### 4.9. Immunofluorescence F-Actin Staining

The cells were seeded onto the glass-bottom of fibronectin (FC010, Millipore, Temecula, CA, USA) coated culture dishes (P35GC-014-C; MatTek, Ashland, MD, USA). Following the 36 h attachment, the cells were fixed with 3.7% paraformaldehyde, and then, permeabilized with 0.1% Triton X-100 in phosphate buffered saline (PBS) at room temperature for 10 min. The cells were washed with PBS and blocked in a 1% bovine serum albumin (BSA)/PBS solution for another 1 h. The F-actin protein expression was revealed by its incubation with Texas Red X-Phalloidin (Invitrogen) and the immunofluorescence was recorded using a confocal microscope (LSM510 Meta, Zeiss, Oberkochen, Germany), as described previously [20]. The F-actin fluorescence intensity was assessed using Zen blue edition software (version 3.2, Zeiss). The intensity profiles were measured along the line from the peripheral to the central of the cells.

### 4.10. Enzyme Linked Immunosorbent Assay

The cells were incubated in a 0.5 mL of a RPMI 1640 medium supplemented with 10% FBS. The IL-6 and WISP1 protein levels of the cell supernatant were measured, respectively, by IL-6 (Catalog #: D6050; R&D Systems, Inc., Minneapolis, MN, USA) and the WISP1 (ELH-WISP1, RayBiotech, Norcross, GA, USA) enzyme linked immunosorbent assay kit, as described previously [20,56]. The protein levels in each sample were adjusted by the concentration of the protein in the whole cell extract, which was measured using a bicinchoninic acid protein assay kit (Pierce Protein Research, Rockford, IL, USA).

### 4.11. Xenograft Animal Model

The male nude mice (BALB/cAnN-Foxn1, 4 weeks old) were purchased from the animal center of the Ministry of Science and Technology, ROC (Taiwan). The PC-DNA and PC-WISP1v1 cells were treated with Gibco™ Versene solution (Thermo Fisher Scientific, Grand Island, NY, USA) to detach, and then washed with a RPMI1640 medium plus 10% FBS. The animals were randomized into two groups and were anesthetized intra-peritoneally. The equal volumes of cells (4.6 × 10^6^/100 μL PBS) were injected subcutaneously on the lateral back wall of each mouse. The tumor volume was measured at three-day intervals using vernier calipers and determined using the following formula: volume = π/6 × larger diameter × (smaller diameter)^2^, as described previously [55]. Following 41 days of inoculation, mice were sacrificed, the tumors were recorded, and the tumor weight was measured. The expressions of WISP1, CXCL5, NDRG1, or IL-6, were determined by immunoblot and RT-qPCR assays, as described above.

### 4.12. Statistical Analysis

The results are expressed as the mean ± standard error (S.E.). The statistical significance was determined with the Student *t*-test and one-way ANOVA using SigmaStat software for Windows version 2.03 (SPSS Inc., Chicago, IL USA). The application of the post-hoc analysis was used to correct for multiple comparisons.

## 5. Conclusions

The transformation of normal cells to the malignant or benign phenotype involves various signal transduction systems. The tumor microenvironment, including the epithelial-stromal crosstalk during initiation progression and metastatic potential of prostate cancer, is still not well-defined. Our results indicated that WISP1 is a stroma-specific protein secreted from the prostate fibroblast cells but not the carcinoma cells in vitro. The prostate fibroblast HPrF cells express two WISP1 isoforms (WISP1v1 and WISP1v2) which both are induced by TNFα treatments. Either WISP1v1 or WISPv2 induces the proliferation, migration, and contraction of human fibroblast HPrF cells in vitro. However, only the ectopic overexpression of WISP1v1 enhances the proliferation and invasion of prostate carcinoma cells in vitro and tumor growth in vivo. Our results indicate that WISP1 affects the cell proliferation, migration, and contraction of fibroblasts in an autocrine manner, and suggest that WISP1 should be regarded as an oncogene in the human prostate cancer.

## Data Availability

Not applicable.

## Supplementary Materials

The supporting information can be downloaded at: https://www.mdpi.com/article/10.3390/ijms231911437/s1.
