# Supplementary material for: WNT1 Inducible Signaling Pathway Protein 1 Is a Stroma-Specific Secreting Protein Inducing a Fibroblast Contraction and Carcinoma Cell Growth in the Human Prostate
Source: Int J Mol Sci. 2022 Sep 28;23(19):11437. doi: 10.3390/ijms231911437 (PMC9570503; doi:10.3390/ijms231911437)
Supplement: Supplementary file 1 [file ijms-23-11437-s001.zip › ijms-1906088-supplementary.pdf]

A

gtaagatgtgcgctcagcagcttggggacaactgcacggaggctgccatctgtgaccccccac
K M C A Q Q L G D N C T E A A I C D P H
cggggcctctactgtgactacagcggggaccgcccgaggtacgcaataggagtgtgtgca
R G L Y C D Y S G D R P R Y A I G V C A
caggtggtcggtgtgggctgcgtcctggatggggtgcgctacaacaacggccagtccttc
Q V V G V G C V L D G V R Y N N G Q S F
cagcctaactgcaagtacaactgcacgtgcatcgacggcgcggtgggctgcacaccactg
Q P N C K Y N C T C I D G A V G C T P L
tgcctccgagtgcgcccccccgcgtctctggtgcccccacccgcggcgcgtgagcatacct
C L R V R P P R L W C P H P R R V S I P
ggccactgctgtgagcagtgggtatgtgaggacgacgccaagaggccacgcaagaccgca
G H C C E Q W V C E D D A K R P R K T A
ccccgtgacacaggagccttcgatgctgtgggtgaggtggaggcatggcacaggaactgc
P R D T G A F D A V G E V E A W H R N C
atagcctacacaagcccctggagcccttgctccaccagctgcggcctgggggtctccact
I A Y T S P W S P C S T S C G L G V S T
cggatctccaatgttaacgcccag
R I S N V N A Q

B

gtaagatgtgcgctcagcagcttggggacaactgcacggaggctgccatctgtgaccccccac
K M C A Q Q L G D N C T E A A I C D P H
cggggcctctactgtgactacagcggggaccgcccgaggtacgcaataggagtgtgtgca
R G L Y C D Y S G D R P R Y A I G V C A
catgctgtgggtgaggtggaggcatggcacaggaactgcatagcctacacaagcccctgg
H A V G E V E A W H R N C I A Y T S P W
agcccttgctccaccagctgcggcctgggggtctccactcggatctccaatgttaacgcc
S P C S T S C G L G V S T R I S N V N A
cag
Q

supplement Figure S1. The sequences and predicative translational proteins of the WISP1v1 (A) and WISP1v2 (B) cDNA fragments synthesize from RT-PCR.
